# Intramedullary spinal cord and filum tumours—long-term outcome: single institution case series

**DOI:** 10.1007/s00701-022-05350-3

**Published:** 2022-09-27

**Authors:** Erling Myrseth, S. Habiba, T. Rekand, H. A. Sætran, S. Mørk, M. Grønning

**Affiliations:** 1 Department of Neurosurgey, Haukeland University Hospital, Bergen, Norway; 2 Department of Neurology, Haukeland University Hospital, Bergen, Norway; 3 Department of Pathology, Haukeland University Hospital, Bergen, Norway; 4 Department of Occupational Medicine, Haukeland University Hospital, Bergen, Norway

**Keywords:** Spinal cord tumour, Long-term outcome

## Abstract

**Background:**

Intramedullary spinal cord tumours are rare and account for about 2–4% of primary CNS tumours. Ependymomas and astrocytomas are most frequent. The aim of this study was to evaluate the long-term neurological outcome, quality of life (QoL), survival, need for additional treatment and frequency of neuropathic pain in a patient group treated at a tertiary university hospital.

**Method:**

Retrospective descriptive study of 52 long-term survivors with intramedullary or filum tumours consenting to participate in this study. Fifty-six operations were performed in 48 patients. Clinical and radiological follow-up period was 113 and 117 months, respectively.

**Results:**

Good neurological outcome (ASIA score D or E, modified McCormick grade 1 or 2) was achieved in 88%. We found two negative prognostic factors in regards of severe disability which were large craniocaudal tumour size (p = 0.004) and histologic verified astrocytomas (p = 0.002). SF-36 results showed significantly lower results on all five subdomains concerning physical function, whereas scores for mental health and role emotional showed no significant differences compared to Norwegian norms.

Ten patients including all astrocytoma patients, one primitive neuroectodermal tumour and three recurrent tumours of filum terminale had adjuvant therapy. None of the patients with intramedullary ependymoma had adjuvant therapy.

Neuropathic pain was present in 54% of patients at the last follow-up.

**Conclusion:**

This series shows that good results can be obtained with surgery for intramedullary tumours, even without perioperative neurophysiological monitoring. Multicentre studies are needed for further evaluation of negative and positive prognostic factors to further improve outcome.

## Introduction

Intramedullary spinal cord tumours are rare and account for about 2–4% of primary CNS tumours [25, 27]. Ependymomas and astrocytomas are most frequent. In Manzano’s series, 50% were ependymomas and 12.5% astrocytomas [25], whereas in Raco’s series, 34% were ependymomas and 42% astrocytomas [27]. In adults, as age increases, the incidence of ependymomas increases, and the incidence of astrocytomas decreases [25]. In addition, some rare tumours like primitive neuroectodermal tumours (PNET), hemangioblastoma, cavernomas, seeding of medulloblastomas and glioblastomas may be found. The age-adjusted annual incidence rate of primary intraspinal tumours in Norway in approximately the same time period was 1.29 per 100,000, increasing to 1.48 per 100,000 in the last quarter of the study period. Spinal cord tumours constituted 31% and 41% of the tumours located in the cauda equine or the spinal nerves. Seventeen percent were ependymomas, and 3% were astrocytomas [35]. In our series, a substantial number of ependymomas were located in the filum terminale. Good quality contrast enhanced magnetic resonance imaging (MRI) can detect the tumour at an early stage, even before the tumour has given rise to disabling symptoms like severe ataxia and para/tetra-plegia. Most patients present with a minor sensory or motor deficit, sometimes in addition to neuropathic pain. The treatment for these tumours is surgical resection, but the fear of poor neurological outcome might have had a great impact on the decision-making as to find the best timing for surgery. However, several publications suggest that best outcome is achieved when surgery is performed early, before significant neurological impairment has developed [17, 19, 28, 36].

The aim of this study was to evaluate the long-term neurological outcome, quality of life (QoL), need for additional treatment and frequency of neuropathic pain in a patient group treated at a tertiary university hospital. We also evaluated the frozen section histopathological result, compared to the final diagnosis, and MRI findings during the follow-up period.

The study is approved by the regional ethical committee (2011/2429 IMSHUS).

## Method

**Fig. 1:**
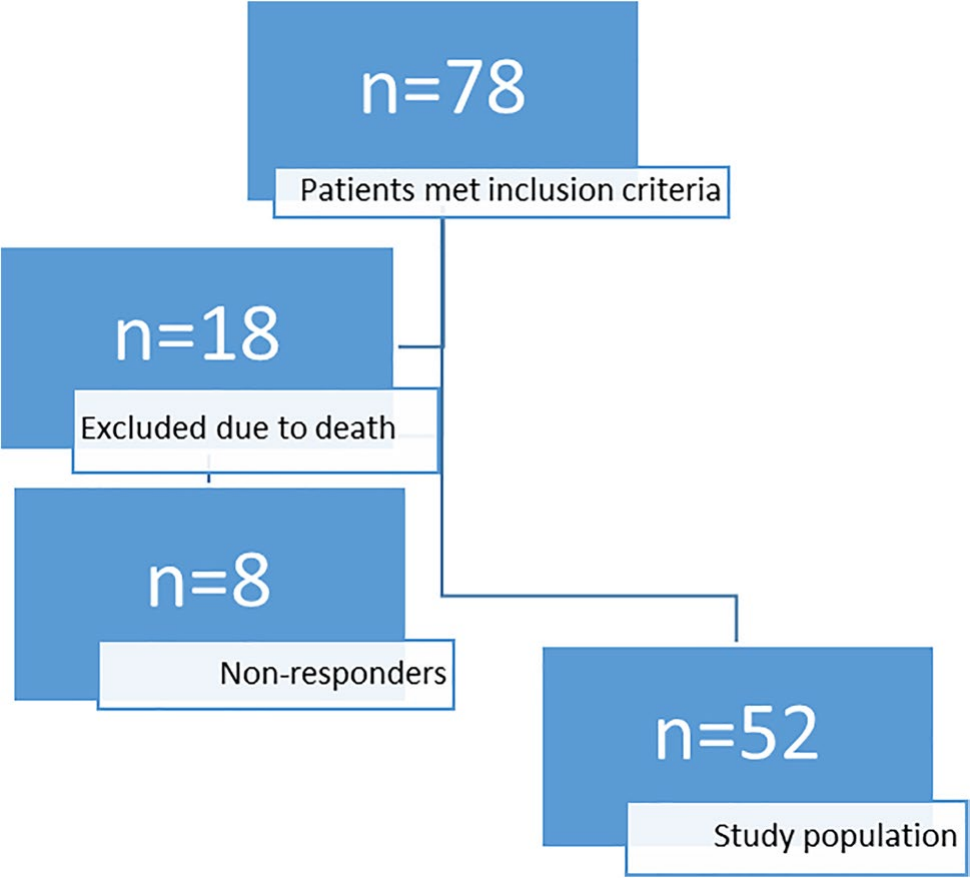
Study population

In this descriptive follow-up study, patients were identified using hospital records searching for the ICD-10 diagnosis D32.1, D33.4, D36.1, C70.1, C72.0 and C72.1 during the years 1991 through 2012. Seventy-eight patients met the requirements of an intramedullary tumour or cyst. Eighteen patients had died before study start. The 60 living patients were invited to participate in the study by mail, and the consent was achieved from 52 patients (87%) (Fig. 1).

Data were collected from patient files and from interviews at the last admission. The patients were examined by one of two neurologists evaluating the ASIA score and McCormick grade at last follow-up and estimating the ASIA score and McCormick grade before treatment based on the patient’s chart. Four patients did not want to come for a clinical follow-up, but consented to the use of the data from their hospital files in the study. Five patients died during the study period before the last study follow-up by a neurologist. Three patients who died due to the spinal tumour were scored according to the best postoperative status. The two patients who died of tumour-unrelated reasons were scored according to the latest clinical follow-up. Fourty-eight patients had a MRI of the spinal column at the last follow-up, and 45 patients filled out questionnaires for QoL (SF-36).

Two experienced pathologists blinded for the primary diagnosis reviewed the histopathological samples. Individual assessments were compared and discrepancies were settled by joint review of the slides in question. The reviewed histopathological diagnoses were compared with the primary diagnosis.

### Statistical analyses

Baseline characteristics with differences between subgroups were assessed with Student’s independent samples *t*-test or chi-squared tests. Statistical significance was p = 0.05. Normal distribution was checked (Q-Q plots), and central tendency presented as means, or medians if skewed. To get a better model-data fit, we dichotomized two variables: thoracic vs other (yes/no) and astrocytoma vs other (yes/no). Additionally, we produced a variable from the combination of ASIA score (A-C) and modified McCormick grade (3–4) to non-ambulant/severe difficulty walking (yes/no). SPSS for Windows version 23.0 was used for all analyses.

## Results

Table 1 shows the population characteristics of the 52 study patients.

**Table 1:** Characteristics of the 52 study patients

	Mean
Male Female	N = 23 N = 29
Age at treatment strategy (years)	40.78
Age at last follow-up (years)	51.30
Symptom duration prior to treatment strategy (months)	18.27
Time from diagnosis to treatment strategy (months)	5.57
Time from treatment strategy to last clinical follow-up (months)	112.80
Time from treatment strategy to last radiological follow-up (months)	117.00
Craniocaudal size of lesion (mm)	50.88
Tumour extent along multiple levels	2.78
Cervical location of tumour	N = 24 (46.2%)
Thoracic location of tumour	N = 9 (17.3%)
Lumbar location of tumour	N = 19 (36.5%)

Four patients with intramedullary tumours having minor or no symptoms were observed clinically for 57–91 (mean 77) months and with MRI scans 68–95 (mean 86) months. These tumours did not grow during the observation period, and the patients had no treatment. Three asymptomatic patients had MRI due to other reasons, which incidentally detected cervical spine ependymomas. Two of these patients had follow-up MRI 6 months later, which showed tumour growth, and both were operated. The third asymptomatic patient had a large tumour in the cranio-cervical junction, and surgery was performed without an observation period.

Initially, all patients except two had estimated ASIA score D or E, and 45 patients had modified McCormick grade 1 or 2a (Tables 2 and 3).

**Table 2:** ASIA score

ASIA		Intramedullary tumours n = 33	Intramedullary tumours n = 33	Filum tumours n = 19	Filum tumours n = 19
		First admission	Last follow-up	First admission	Last follow-up
ASIA score	A	0	1	0	0
	B	0	1	0	0
	C	2	2	0	0
	D	19	25	8	10
	E	12	4	11	9

**Table 3:** Modified McCormick grade

Modified McCormick grade		Intramedullary tumours n = 33	Intramedullary tumours n = 33	Filum tumours n = 19	Filum tumours n = 19
		First admission	Last follow-up	First admission	Last follow-up
Grade	1	18	20	16	17
	2a	10	6	1	0
	2b	1	2	2	2
	3	4	3	0	0
	4	0	2	0	0

Surgery was performed 0–70 (median 2) months after the diagnosis. Six patients had observation time of more than 8 months before surgery. All patients were operated in the prone position with laminectomy and midline opening of the dura and spinal cord. As judged by the surgeon, 17 of a total of 18 intramedullary ependymomas, one of a total of 6 astrocytomas and 12 of a total of 19 myxopapillary ependymomas of filum terminale were totally resected at the primary procedure. Two patients with astrocytomas and the patient with a cyst in conus medullaris had only biopsy, and one patient with a cystic ependymoma in the cervical spinal cord had biopsy and a very limited resection.

Fifty-six operations were performed in 48 patients. Seven patients had two procedures and one patient had a total of three procedures, two due to residual tumour (one astrocytoma and one myxopapillary ependymoma) and five due to tumour recurrences (three astrocytomas and two large myxopapillary ependymomas of filum terminale). Most of the procedures for pure intramedullary tumours (n = 21, 79%) were performed by the same surgeon (EM), whereas four different surgeons performed the other procedures.

Intraoperative electrophysiological monitoring was not available in this series, except for two patients where somatosensory evoked potentials (SEP) and motor evoked potentials (MEP) were used.

### Neurological outcome

Tables 2 and 3 show the neurological outcome compared to the neurological function at first admission assessed by the ASIA score and McCormick grade. A good neurological outcome defined as ASIA score D + E or modified McCormick grade 1–2 was achieved in 88% and 85%, respectively, of intramedullary tumour patients and in all filum tumour patients. Eight patients changed from ASIA E to D due to sensory deficits. Two patients deteriorated from ASIA D + E to C, and only one patient lost more than one level in ASIA score (from C to A). According to McCormick grading, one patient with an intramedullary tumour deteriorated from grade 1 or 2 to grade 3 or 4. The 12 patients with preoperative ASIA grade E were all able to walk independently, whilst 3 out of 19 patients (15.8%) with ASIA grade D needed at least aid like crutches. At the last follow-up, four out of 33 patients (12.1%) with intramedullary tumours were dependent of walking support or wheelchair.

Only one patient (4.2%) with a tumour in the cervical cord needed walking support, whilst one-third of the patients with tumours in the thoracic cord or filum were dependent of walking aids postoperatively. Only one of the 18 patients with intramedullary ependymomas was dependent of postoperative walking support, whilst half of the six patients with astrocytomas were so (p = 0.002).

For patients with filum ependymoma, the neurological function did not change significantly.

The risk factors for a severe outcome for pure intramedullary tumours were evaluated by computing a variable that combined both the ASIA score (A, B and C) in addition to McCormick grading (3 and 4) for better data fit. These patients were evaluated as severely disabled and needed either walking aid or wheel chair. In a univariate analysis, large craniocaudal tumour size and histologic verified astrocytomas were identified as negative prognostic factors, p = 0.004 and p = 0.002, respectively. Other clinically relevant factors as cystic tumours, symptom duration, location, contrast enhancement and time from diagnosis to surgery did not show any negative prognosis (Table 4). However, when including the prognostic factors with a statistical significance in a multivariate regression analysis, we could not find any significant risk factor.

**Table 4:** Evaluation of possible negative prognostic factors for intramedullary tumours

Possible prognostic factors	Study population of intramedullary tumours n = 33	Walking aid or need for wheel chair n = 4	Significance (*p* value)
Mean symptom duration prior to treatment strategy (months)	7.77	7.75	0.998
Mean time from diagnosis to treatment strategy (months)	21.33	40.75	0.095
Craniocaudal size of lesion (mm)	53.60	121.33	**0.004**
Tumour extent along multiple levels	3.15	4.50	0.130
Thoracic vs other locations n (%)	4	2	0.078
Asctrocytoma histology vs other, n (%)	3	3	**0.002**
Contrast enhancement, n (%)	22	2	0.160
Cystical lesions vs solid, n (%)	17	2	0.744

Significance of ≤ 0.05

### Quality of life

**Fig. 2:**
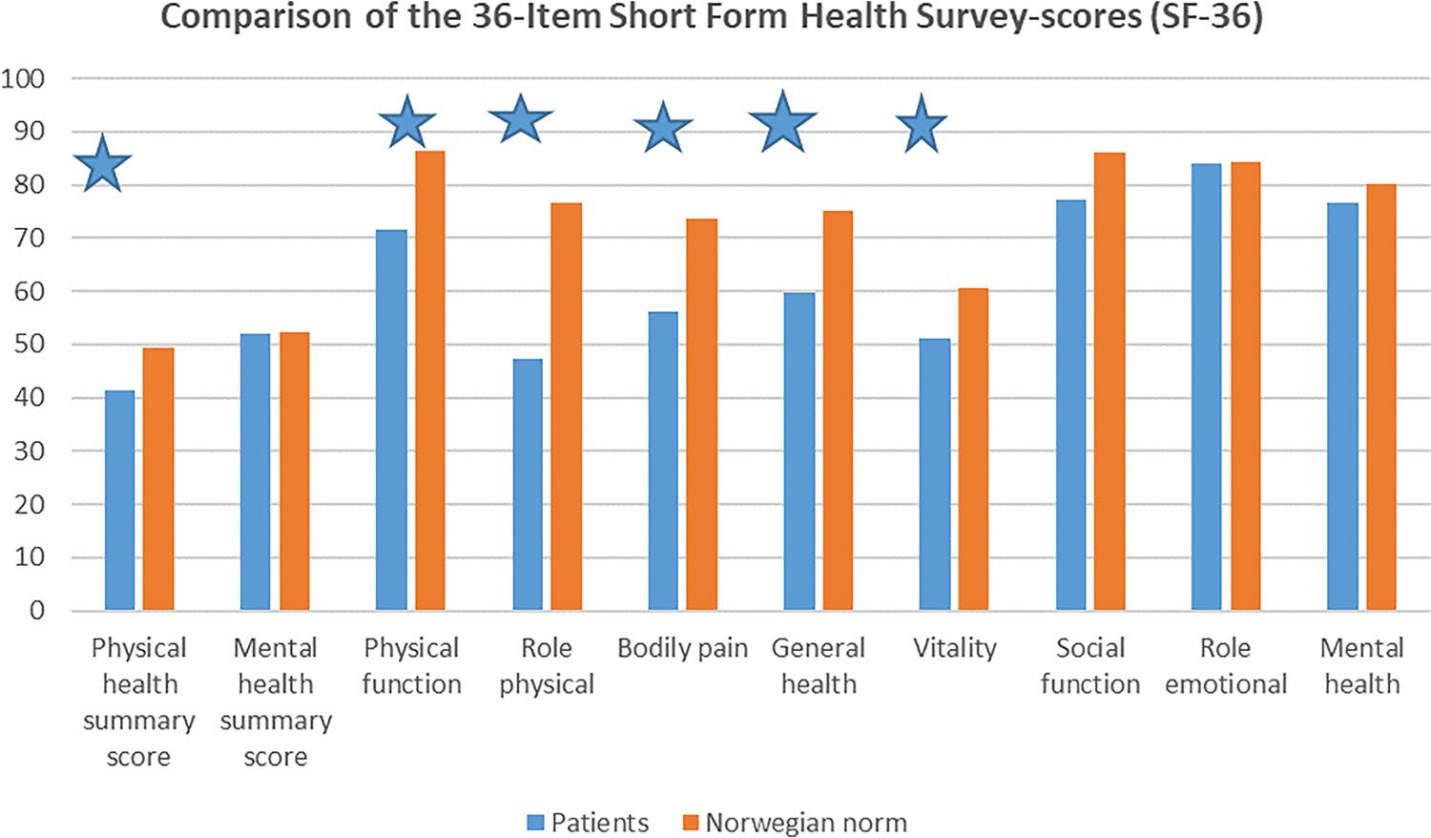
SF-36

According to the SF-36 questionnaire, the 45 eligible patients scored significantly lower than the Norwegian norms on physical health summary score, but not on mental health summary score. Patients scored significantly lower on all five subdomains concerning physical health (physical function, role physical, bodily pain, general health and vitality), whereas scores for mental health and role emotional showed no significant differences (Fig. 2 and Table 5).

**Table 5:** Comparison of the 36-Item Short Form Health Survey-scores (SF-36)

	Patients n = 45		Norwegian norm* n = 5396		
	Mean	(SD)	Mean	(SD)	p
Physical health summary score	41.45	(12.32)	49.49	(10.16)	< 0.001
Mental health summary score	51.93	(8.48)	52.19	(9.08)	0.8482
Physical function	71.58	(28.88)	86.44	(20.42)	< 0.001
Role physical	47.40	(43.53)	76.64	(37.39)	< 0.001
Bodily pain	56.08	(28.28)	73.62	(25.83)	< 0.001
General health	59.83	(26.15)	75.25	(21.72)	< 0.001
Vitality	51.17	(21.04)	60.72	(20.61)	0.002
Social function	77.34	(22.87)	86.27	(21.18)	0.0049
Role emotional	84.03	(32.97)	84.23	(31.67)	0.9664
Mental health	76.77	(12.83)	80.27	(15.47)	0.1303

^*^ Measurement properties and normative data for the Norwegian SF-36: results from a general population survey, Garrat AM, Health Qual Life Outcomes. 2017 Mar 14;15(1):51

### Histopathology

The histopathological diagnosis was assessed according to the WHO classification of tumours of the central nervous system version 2007, and the numbers of tumours are shown in Table 6. Two ependymomas were subependymoma WHO grade 1. Two astrocytomas were graded as WHO grade 2, two grade 2–3, one grade 4 and one uncertain. The other tumours were one ganglioglioneurocytoma, one PNET, one paraganglioma, one hemangioblastoma WHO grade 1 and one cyst in conus.

**Table 6:** Histopathological tumour type

Histopathology	Frequency	Percent
Astrocytoma	6	11.5
Ependymoma	18	34.6
Filum ependymoma	19	36.5
Other	5	9.6
Not operated	4	7.7
Total	52	100

Two cases were given a new diagnosis based on the re-examination. One ependymoma WHO grade 2 was reviewed as a myxopapillary ependymoma WHO grade 1, and one myxopapillary ependymoma was classified as ependymoma WHO grade 2. For 7 cases, no slides were available for re-examination, and their primary diagnosis was kept.

Frozen section diagnosis was available in 35 cases and was identical to the final histopathological diagnosis in 22 cases (63%). A correct frozen section diagnosis was made in 19 out of 22 ependymomas (86%) and 3 out of 6 astrocytomas (50%). None of the four patients with “other” tumours had correct frozen section diagnosis.

### MRI

In the preoperative evaluation of MRI scans, most of the results were reported as “tumour”, but not specified any more. In only ten cases, the tentative radiological diagnosis was consistent with the histopathological diagnosis.

**Fig. 3:**
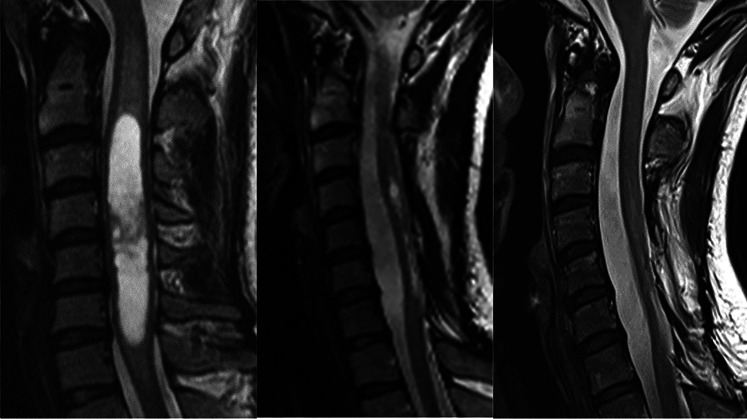
Cystic ependymoma. T2 weighted MRI preoperatively, 1 year and 7 years postoperatively

**Fig. 4:**
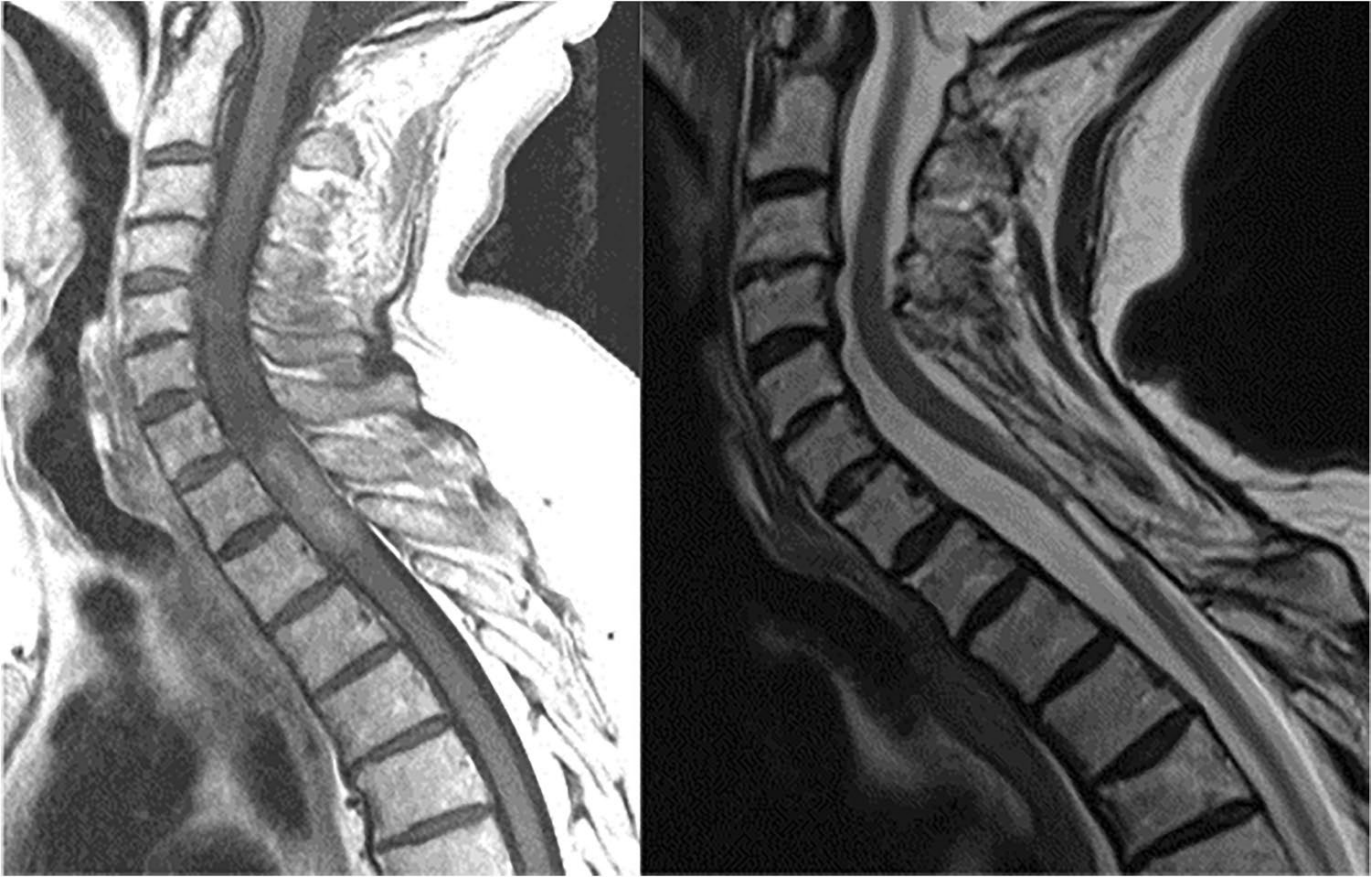
Ependymoma. MRI T1 preoperatively, T2 10 years postoperatively

**Fig. 5:**
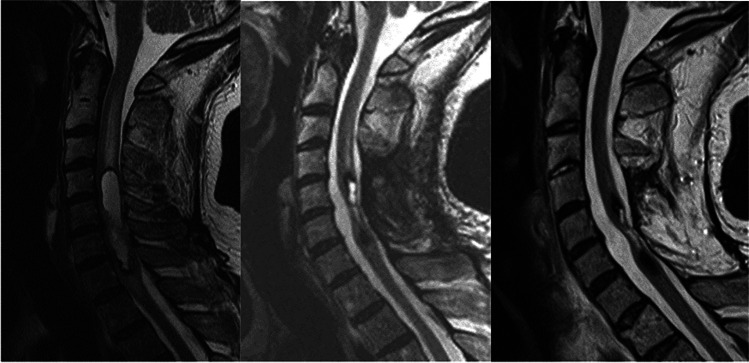
Intramedullary ependymoma. T2 weighted MRI preoperatively, 6 months and 5.5 years postoperatively

**Fig. 6:**
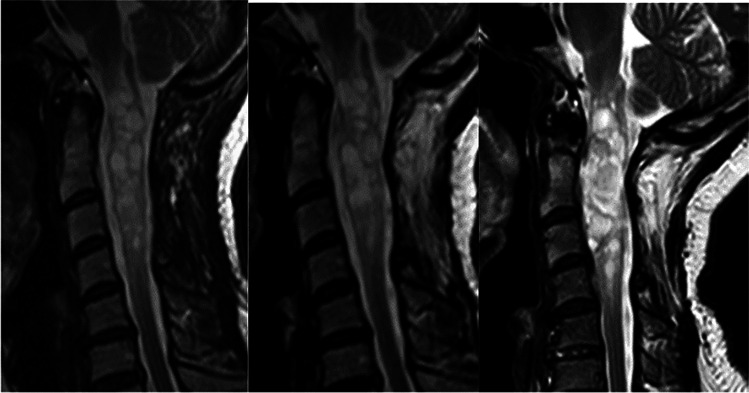
Intramedullary cystic ependymoma. T2 weighted MRI preoperatively, and 3 and 11 years postoperatively

The last MRI scan of the 48 treated patients at an average of 117 months (18–260) postoperatively showed that 14 patients had no visible tumour (Figs. 3 and 4), 30 patients had unchanged postoperative signs (Fig. 5), three patients had residual or remnant tumours (Fig. 6) and one patient had an increasing cyst and atrophy of the spinal cord.

### Adjuvant treatment

Ten patients had adjuvant treatment. Five patients had postoperative radiotherapy after the primary surgery, and the other five patients had radiation in combination with chemotherapy for recurrent tumour. These ten patients were six astrocytoma patients, two recurrent and one residual myxopapillary ependymoma of filum terminale and one PNET. None of the patients with intramedullary ependymoma had adjuvant therapy.

### Pain

Twenty-eight patients (54%) suffered from neuropathic pain at the last follow-up, 18 patients (54.5%) with tumours in the spinal cord and 10 patients (52.6%) with filum tumours. Unfortunately, the patients’ charts were insufficient regarding preoperative pain.

### Survival

Five patients died during the study period, 60–247 months after the surgery. Three patients had astrocytoma grade 2, 3 and 4, respectively, and died due to the tumour. Two of them had more than one surgical procedure, and both had other malignant disease (glioblastoma multiforme and non-Hodgkin’s disease). Two patients with ependymoma of filum terminale, one with grade 1 and one with grade 2, had unknown cause of death.

## Discussion

Intramedullary spinal cord tumours are most challenging to treat, with a significant risk of developing neurological deficit independent of treatment strategy. The natural course of ependymomas and even low-grade astrocytomas is usually rather benign with slowly progression of neurological symptoms over years, which traditionally has favoured a wait-and-see strategy or a conservative strategy with biopsy and spinal and dural decompression, and eventually adjuvant radiotherapy and/or chemotherapy [10, 11, 30]. However, during the last two or three decades, advances in surgical resection techniques with ultrasound aspirators, as well as improved electrophysiological monitoring and interpretation of signals, have advocated a more surgical approach to these tumours [8, 10, 11, 30]. Surgery has been shown to be least hazardous when the neurological deficit is minimal or non-existent [9–11, 17, 23, 24].

In this series, seven patients with tumours indicating ependymomas had no or minor symptoms. Six patients had serial follow-up MRI scans showing non-growing intramedullary tumours in four of them. They had no treatment, which is in accordance with the recommendations of Harrop et al. [17]. However, Aghakhani et al. [1] recommend that surgery should be considered carefully also to ependymoma patients with no or minor symptoms. Two of our asymptomatic patients had growing tumours, and one patient had a large tumour in the craniocervical junction, and these three patients were operated.

Most attention has been paid to preservation of motor function after intramedullary tumour resection. However, merely opening of the spinal cord to expose the tumour can lead to dorsal column dysfunction (DCD) including numbness and painful dysesthesias, as well as reduced joint sensibility, causing gait disturbances even with intact motor function. This might be the most debilitating postoperative morbidity experienced by these patients. New or increased DCD was seen in 43.6% of Manzano’s patients [25] and 67% in Halvorsen’s series [16].

The most important factor for achieving complete resection is finding of a cleavage plane between the tumour and spinal cord [13, 14]. In contrast to intramedullary astrocytomas, intramedullary ependymomas are usually well defined with a cleavage plane between the tumour and the spinal cord, making a complete resection possible. This is also the goal in ependymoma surgery. However, in a meta-analysis of grade 2 ependymomas, Sun et al. [31] did not find any significant difference in progression free survival and overall survival between age, sex, tumour length, total resection group, subtotal resection group and biopsy and decompression group or chemotherapy. Therefore, surgical resection must be safe in respect of neurological function. It is usually preferable to leave a small area of residual tumour rather than to risk neurological morbidity [30].

The neurological outcome in this series is encouraging and comparable with other series [1, 5, 15]. For intramedullary tumours, some deterioration mostly due to sensory deficits is expected, whilst the neurological outcome for filum tumours most often is equal to the preoperative status.

Not surprisingly, SF-36 showed a significant reduction in physical health subdomains, but not in mental health domains, compared to Norwegian norms. Similar results have been found in patients with spinal cord injury [4]. In spite of neurological deficits, the patients seem to cope with their disability. However, one patient who was quite satisfied with the treatment reported very low scores on physical health domains in spite of minor symptoms and no residual tumour.

Neuropathic pain is a common complaint both pre- and postoperatively in patients with intramedullary tumours. The pain fibres are crossing in the spinal cord and have a close relationship to the central canal where the origin of ependymomas is located. In our series, postoperative neuropathic pain occurred in 54% of the patients as compared to 22% in Klekamp’s series [19]. Surprisingly, postoperative neuropathic pain was equally frequent in patients with intramedullary tumours and myxopapillary tumours of the filum terminale.

In Klekamp’s series, syringomyelia and preoperative presence of neuropathic pain appeared to be the strongest predictors of a postoperative neuropathic pain syndrome, and pain was more common in type A tumours (displacing tumours) compared to type B tumours (infiltrative tumours).

A correct frozen section histopathological answer will facilitate the decision-making for the surgeon, as astrocytomas usually grow infiltrating and do not have a clear surgical plane making complete resection possible. Due to the inherent inaccuracy of attempted frozen section diagnosis at the time of operation, Cooper and Epstein [9] meant that frozen section diagnosis was not helpful. In contrast to this, Hongo et al. [18] found that the frozen section and/or dissection plane helps guide neurosurgeons. The frozen-section diagnosis and final permanent-section diagnosis in their series agreed in 23 (72%) of 32 cases of ependymoma, and in 12 (71%) of 17 cases of astrocytoma. Our results were 86% and 50%, respectively.

Several papers report of the usefulness of electrophysiological monitoring in improving neurological outcome [7, 8, 20–22, 26], but none of the studies are RCTs comparing surgery with or without electrophysiologic monitoring. Perioperative electrophysiological monitoring using somatosensory evoked potentials (SEP), motor evoked potentials (MEP) and D-wave recording is now regarded mandatory in surgery for intramedullary spinal cord tumours. Electrophysiological monitoring is a tool to help the surgeon, but cannot replace the skill of the surgeon. In this series, perioperative neurophysiological monitoring was not used, except for two patients. Nevertheless, our results show that good outcome can be achieved also without perioperative monitoring.

Some of the ependymoma patients where tumours were totally resected as judged by the surgeon had follow-up MRI that showed some contrast enhancement that did not change over years (Fig. 5). This might be very small remnants, or contrast enhancement in the tumour bed due to damage of blood-cord barrier. Nevertheless, this finding carries a good prognosis. Even known small remnants seem to be unchanged over many years [1].

Even if it is well accepted that volume matters, our fairly good outcomes are achieved in a small volume centre. However, most of the patients are handled by one experienced senior neurosurgeon, which may reduce the possible negative effect of few procedures.

The role of postoperative radiotherapy for astrocytomas seems well established, but totally resected ependymomas do not need radiotherapy [2, 6, 11, 23, 30, 33]. For recurrent tumours or growing remnants, surgery should be offered [6, 30]. For residual or recurrent high-grade (WHO grades 3 and 4) tumours, radiotherapy is recommended, and chemotherapy is also a treatment option [17]. Myxopapillary ependymoma (MPE) has a generally benign clinical course [2]. However, MPE of the paediatric age group is reported to be more aggressive with tendency for more dissemination (up to 80%) [3, 12].

Using a univariate analysis, we found that large craniocaudal tumour extension and astrocytoma were negative prognostic factors. However, using multivariate regression analysis, these could not be confirmed. This is probably due to the small data sample, as others have found large tumour extension as negative prognostic factor [15, 34].

The weakness of this study is the retrospective design and the small patient population.

Due to the infrequent number of patients with intramedullary spinal cord tumours, to our knowledge, no prospective studies are published, and the numbers of patients in the existing retrospective papers are usually rather small. The need for multi-centre prospective studies is therefore obvious.

## Conclusion

This series show that good results are achievable with surgery for intramedullary tumours, even without perioperative neurophysiological monitoring. We recommend that patients with intramedullary tumours without neurological symptoms should undergo a wait-and-see follow-up with serial MRI scans, and surgical resection if tumour growth is detected. For patients with tumour-related symptoms, surgical resection should be recommended, unless there is contra-indications. However, dorsal column dysfunction is likely to occur, and patient’s information in this regard should be emphasised prior to surgery. During recent years, the use of multimodal neurophysiological monitoring has shown encouraging results regarding neurological outcome and is now considered mandatory in surgical treatment of intramedullary tumours.
